# Chronic disease and recent addiction treatment utilization among alcohol and drug dependent adults

**DOI:** 10.1186/1747-597X-6-28

**Published:** 2011-10-18

**Authors:** Sharon Reif, Mary Jo Larson, Debbie M Cheng, Donald Allensworth-Davies, Jeffrey Samet, Richard Saitz

**Affiliations:** 1 Institute for Behavioral Health, Heller School for Social Policy and Management, Brandeis University, 415 South Street, MS035, Waltham, MA 02454, USA; 2 Boston University School of Public Health, 801 Massachusetts Avenue, Boston, MA 02118, USA; 3 Boston University School of Medicine, 801 Massachusetts Avenue, Boston, MA 02118, USA; 4 Boston Medical Center, 801 Massachusetts Avenue, Boston, MA 02118, USA

**Keywords:** addiction, substance abuse, substance abuse, treatment, medical care, chronic disease

## Abstract

**Background:**

Chronic medical diseases require regular and longitudinal care and self-management for effective treatment. When chronic diseases include substance use disorders, care and treatment of both the medical and addiction disorders may affect access to care and the ability to focus on both conditions. The objective of this paper is to evaluate the association between the presence of chronic medical disease and recent addiction treatment utilization among adults with substance dependence.

**Methods:**

Cross-sectional secondary data analysis of self-reported baseline data from alcohol and/or drug-dependent adults enrolled in a randomized clinical trial of a disease management program for substance dependence in primary care. The main independent variable was chronic medical disease status, categorized using the Katz Comorbidity Score as none, single condition of lower severity, or higher severity (multiple conditions or single higher severity condition), based on comorbidity scores determined from self-report. Asthma was also examined in secondary analyses. The primary outcome was any self-reported addiction treatment utilization (excluding detoxification) in the 3 months prior to study entry, including receipt of any addiction-focused counseling or addiction medication from any healthcare provider. Logistic regression models were adjusted for sociodemographics, type of substance dependence, recruitment site, current smoking, and recent anxiety severity.

**Results:**

Of 563 subjects, 184 (33%) reported any chronic disease (20% low severity; 13% higher severity) and 111 (20%) reported asthma; 157 (28%) reported any addiction treatment utilization in the past 3 months. In multivariate regression analyses, no significant effect was detected for chronic disease on addiction treatment utilization (adjusted odds ratio [AOR] 0.88 lower severity vs. none, 95% confidence interval (CI): 0.60, 1.28; AOR 1.29 higher severity vs. none, 95% CI: 0.89, 1.88) nor for asthma.

**Conclusions:**

In this cohort of alcohol and drug dependent persons, there was no significant effect of chronic medical disease on recent addiction treatment utilization. Chronic disease may not hinder or facilitate connection to addiction treatment.

## Introduction

Chronic medical diseases are long-lasting conditions, often progressive, and often controllable with continuing care and behavior change. In an era of increasing health care costs [1], chronic disease stands out as a major contributor [2] with alcohol and drug use disorders playing an exacerbating role [3-5]. To improve population health and reduce U.S. health care costs, particular attention must be paid to the role of chronic diseases, including medical, psychiatric, and substance use disorders.

Substance use disorders (SUDs) are prevalent in about 9% of the U.S. population, but only 10% of those individuals access addiction treatment [6]. Although medical needs were not explicitly mentioned in this household survey as a reason for not accessing addiction treatment [6], medical needs may interfere with other priorities. For example, chronic medical conditions could cause functional limitations that preclude access to other health care. Interference from pressing medical conditions may thus contribute to perceived unimportance of addiction treatment among the 94% of people [6] deemed in need but who reported they did not need it. To enhance care of both chronic medical diseases and addiction, it is important to better understand their relationships.

More than 40% of the U.S. population is estimated to have at least one chronic medical disease [2,7], with prevalence higher for women and increasing dramatically with age [7]. Chronic medical disease is related to 70% of all U.S. deaths [2], and accounts for more than 75% of health care costs [2] and high out of pocket health care spending [7]. Diabetes, hypertension, and high cholesterol comprise nearly a third of all chronic conditions [7], and just seven conditions account for about one-fourth of annual outpatient visits and hospital discharges [8].

Chronic disease may have unexpected consequences on healthcare utilization, potentially reducing treatment of unrelated disorders, perhaps due to less time available, complexity of navigating multiple problems at once, or a preference to focus on the most troublesome problem [9]. In practice, however, perhaps due to more frequent interaction with medical providers, mental disorders are more often detected among patients with chronic medical diseases than among other patients [10]. Physician awareness of a substance abuse problem also is more likely when the person has episodic or chronic medical illness, however SUDs remain undiagnosed by primary care physicians in nearly half of patients seeking addiction treatment [11].

It is well documented that SUDs cause or exacerbate certain medical conditions and increase their costs [3,12-16]. Incidence for many chronic medical diseases, including hypertension, diabetes, asthma, chronic liver disease, chronic obstructive pulmonary disease (COPD), pain, and stroke is elevated, and for some disorders more than doubled, among people with SUDs [17-20]. Many more conditions are worsened by, have their management impaired by, or are attributable to SUDs [21]. Chronic conditions are prevalent even in younger individuals with SUDs [22]. These findings highlight the importance of medical management as an essential element of addiction treatment [23] particularly in early recovery [24].

Several examples of this complex interplay are reported in the literature. Alcohol use disorders increased medical illness complexity in a primary care population, even after controlling for medical morbidity, initial primary care utilization, and current medical care utilization [25]. Alcohol use disorders also were related to inconsistent attendance and less complex services for the chronic medical disease, suggesting that medical care may be less accessible for the comorbid population, who may require an increased focus on the complexities of care [26]. Among patients receiving diabetes care in the Department of Veterans Affairs, those who also had SUDs or mental illness had more diabetes complications [27]. Although SUDs have been linked to higher utilization of primary, specialty, and emergency medical services [25], there are few studies of how presence of complex medical disorders affects addiction treatment utilization.

Chronic medical diseases require regular and longitudinal care and self-management for effective treatment [28-30]. When chronic diseases include SUDs, care and treatment of both the medical and addiction disorders may affect access to care and the ability to focus on both conditions. An integrated approach could lead to effective treatment for all conditions [29], yet little is known about how chronic medical disease affects addiction treatment utilization. Chronic disease could cause functional limitations that interfere with an individual's ability to access addiction treatment, or alternatively could increase interaction with the health care system and perhaps addiction treatment. Involvement in addiction treatment could lead to linkage with medical care and effective attention to a chronic medical problem. The objective of this paper is to evaluate the association between the presence of chronic medical disease and recent addiction treatment utilization among adults with substance dependence.

## Methods

### Data Source and Sample

Data are from baseline interviews with 563 alcohol and/or drug-dependent adults enrolled in the AHEAD (Addiction Health Evaluation And Disease management) study, a randomized controlled trial testing the effectiveness of a chronic disease management program for substance dependence in primary care.

Study recruitment occurred from a free-standing inpatient detoxification unit (74% of enrolled subjects), primary care clinics and the emergency department in the urban medical center where the study was located (10%) and the community by advertising on buses and in newspapers (16%); subjects were recruited between September 2006 and September 2008.

Adults were eligible if they had (1) a diagnosis of current alcohol or drug dependence as assessed by the Composite International Diagnostic Interview Short Form (CIDI-SF) [31]; (2) past 30 day drug use or heavy alcohol use (defined as ≥4 standard drinks for women, ≥5 for men at least twice; or >14 drinks per week for women, >21 drinks per week for men, in an average week in the past month); and (3) were willing to establish or continue primary medical care at the study location. Subjects were excluded if they could not provide contact information for tracking purposes; were not fluent in English or Spanish; had specific plans to leave the area that would preclude in-person research assessments; were pregnant; or had a Mini-Mental State Examination [32] score <21.

Trained research associates administered the standardized baseline research assessment and assured confidentiality. Data collected include sociodemographics, substance use and related problems, anxiety, depression, health questions, and medical and addiction service use. Subjects were compensated for their study participation after completing baseline study procedures.

The Institutional Review Board approved the study. Additional privacy protection was secured by a Certificate of Confidentiality issued by the Department of Health and Human Services.

### Chronic Disease and Other Medical Condition Measures

Subjects were asked if a doctor had ever told them they had any of a series of medical conditions. A number of these conditions were included on a chronic disease comorbidity questionnaire validated by Katz et al. [33], which is a self-report version of the medical record-based Charlson Comorbidity Index [34]. The Katz questionnaire includes myocardial infarction, congestive heart failure, peripheral vascular, stroke or other cerebrovascular disease, COPD, ulcer disease, diabetes, renal, connective tissue disease, and other conditions (dementia, liver disease, leukemia, lymphoma, cancer, AIDS). Scoring replicates the Charlson Comorbidity Index, which empirically weighted severity by disease (e.g., cerebrovascular = 1, moderate or severe renal disease = 2, AIDS or metastatic solid tumor = 6), based on 1-year adjusted relative risk for mortality [34]. The comorbidity score is the weighted sum of the specified conditions.

The main independent variable, chronic disease status, is a 3-category variable which classified comorbidity scores as: none; only one condition of lowest severity (Katz = 1); or, more than one condition of lowest severity or one or more condition(s) of higher severity (Katz > 1). Asthma was considered in separate secondary analyses, as an example of a specific symptomatic condition, selected due to its high prevalence in the sample; it is also included in the Katz questionnaire, as part of COPD, with a weight of 1. Asthma is a common comorbidity among urban young to middle-age adults, reflecting the age range common for addiction, whereas other common chronic diseases such as heart disease are less common in young populations. Two other common chronic conditions in this population, hepatitis and hypertension, were prevalent but are often asymptomatic to the patient, and thus were not evaluated in the current analyses.

Additional secondary independent variables were used to evaluate whether broader health status significantly predicted addiction treatment utilization: the single item self-report health status from the SF-12 [35] (excellent/very good/good, fair/poor) and physical health-related functioning and quality of life as determined by the SF-12 Physical Component Score (PCS) (continuous 0-100 scale).

### Addiction Treatment Utilization Measures

The primary outcome was any addiction treatment based on self-report, defined as any treatment for addiction (excluding detoxification) in the 3 months prior to study enrollment in any of the following: residential program for alcohol or drug treatment; outpatient full-day/partial hospital program; outpatient with a psychiatrist, other doctor, or other healthcare professional (e.g., counselor); or by taking medication to prevent drinking or drug use. Two secondary outcomes were the counts of outpatient addiction treatment days (at a full-day/partial hospital or with a psychiatrist, other doctor, or other healthcare professional including counselors) and nights in a residential program for alcohol or other drug treatment in the prior 3 months based on self-report at the baseline assessment. These measures were adapted from the COMBINE study Form 90 [36]. Self-report is known to be a reliable way of obtaining treatment utilization information among people with substance use disorders [37,38].

### Covariates

Demographic variables included age, gender, and race/ethnicity. Current type of substance dependence is a 3-category variable indicating either alcohol dependence with heavy alcohol use in the past 30 days, any drug dependence with drug use in the past 30 days, or both. Alcohol and drug dependence were determined using the CIDI-SF alcohol and drug modules [31]; heavy alcohol use in the past month was indicated by 4 or more drinks per day or 15 or more drinks per average week for women, and 5 or more drinks per day or 22 or more drinks per week for men. The regression models included other variables that may affect addiction treatment utilization: recruitment from a detoxification program (versus from medical care or the community), criminal justice involvement (any arrest, probation, parole, pretrial release, diversion program or incarceration in the past 3 months), homeless (any time living in a shelter or on the streets in the past 3 months), unemployed (usual pattern past 3 months), having health insurance (including Medicaid), current smoker, and anxiety in the past week (Beck Anxiety Index [39] scores: low = 0-21, moderate = 22-35, or high = 36 or higher).

### Statistical Methods

Secondary data analysis of the AHEAD study baseline data was conducted. Descriptive statistics were obtained and Spearman correlations were used to evaluate potential collinearity between independent variables and covariates. No pair of variables included in the same regression model was highly correlated (r > 0.40). Multivariate regression models were used to evaluate the effect of presence of chronic disease on addiction treatment utilization, adjusting for covariates defined above.

The primary outcome of any addiction treatment was modeled using logistic regression, with odds ratios and 95% confidence intervals calculated. The distributions for outpatient addiction treatment days and residential nights, which are count variables, were skewed with many zeros and long tails, thus models assuming normality were inappropriate and overdispersed Poisson regression models were used [40]. The Pearson chi-square correction was used to account for overdispersion. The magnitudes of association between measures of chronic disease and utilization were quantified for the Poisson models using incidence rate ratios (IRRs) with 95% confidence intervals. IRR is interpreted as the ratio of the number of outpatient days (or residential nights) for the exposed group of interest (e.g., any chronic disease) versus the reference group (no chronic disease). The null value of no association for the IRR is equal to 1. An IRR >1 indicates that chronic disease is associated with more treatment utilization.

Multivariate models were fit separately for each dependent variable (any addiction treatment, outpatient days, and residential nights) and for each key independent variable (chronic disease, asthma, health status, and PCS). The primary analyses using the Katz score included 498 subjects with complete data on outcomes, the main independent variable, and covariates; secondary models included the same 498 subjects. Power calculations assumed that 27% of subjects without chronic disease utilized addiction treatment (based on the observed data), giving our study approximately 80% power to detect an odds ratio as small as 1.9 for those with one chronic disease of lowest severity and an odds ratio as small as 2.1 for those with one chronic disease of higher severity or multiple chronic diseases. Thus, power was sufficient for effects that were clinically meaningful. All analyses were conducted using two-sided tests and a significance level of 0.05, using SAS software (version 9.1; SAS Institute, Cary, NC).

## Results

### Baseline Characteristics

Of 2,018 potential subjects screened, 650 subjects were eligible, and 563 subjects were enrolled. Enrolled subjects were similar to unenrolled subjects in terms of gender, age and race/ethnicity. Baseline sociodemographic characteristics are noted in Table 1, as are prior healthcare and addiction treatment utilization.

**Table 1:** Baseline Characteristics (n = 563)

	n	%
Age	Mean = 38.2, range 18-67	

Male	409	73

Race		
Black/African American	185	33
White	272	48
Other	106	19

Hispanic or Latino	76	14

Homeless (past 3 mo)	332	59

Unemployed (past 3 mo)	246	44

Has any insurance	446	79

Criminal justice involvement past 3 mo		
None	315	56
Probation	130	23
Pretrial release	63	11
Other/Missing	55	10

Substance dependence		
Alcohol dependent & recent heavy use	98	17
Drug dependent & recent use	150	27
Both	315	56

Substance reported as the major problem		
Alcohol to intoxication	142	25
Heroin	208	37
Cocaine	81	14
Poly-substance or other drugs	132	23

Recruitment site		
Detoxification unit	416	74
Ambulatory/outpatient/ER	59	10
Community/other	88	16

Current smoker	493	88

Beck anxiety score		
Low	220	40
Moderate	164	30
Severe	166	30

Prior healthcare utilization past 3 mo		
Any emergency room	304	54
Any non-addiction outpatient	242	43

Addiction treatment utilization past 3 mo		
Any (excluding detoxification)	157	28
Outpatient days	97	17
Residential nights	70	12

### Addiction Treatment

At baseline, 28% of subjects had been in addiction treatment in the prior 3 months, excluding detoxification (Table 1). The 97 patients (17%) who had outpatient treatment in the prior 3 months attended a median of 5 days (interquartile range 3-13). The 70 patients (12%) with residential treatment in the past 3 months attended a median of 23 nights (interquartile range 8-45).

### Chronic Disease, Physical Health and Other Medical Conditions

Overall, two thirds of subjects had no chronic disease, 20% had one chronic disease of lower severity, and 14% had one of higher severity or multiple diseases (Table 2). One third of subjects reported their health status to be only poor or fair. The mean Physical Comorbidity Score is 41.7 (SD = 8.4) (the general population norm is 50 [35]).

**Table 2:** Chronic Disease and Other Medical Conditions (n = 563)

	n	%
**Summary measures**:		

Chronic disease (Katz)		
None	373	67
One, of lower severity only	109	20
One of higher severity or multiple conditions	75	14

Health status (self-reported)		
Excellent	48	9
Very Good/Good	331	59
Fair/Poor	184	33

Physical Comorbidity Score (SF-12)	mean = 41.7, range 17-60	

**Has a doctor ever told you that you had:**^**1**^		

Hepatitis	181	32

Hypertension*	120	21

Asthma*	111	20

Skin infections	74	13

Pneumonia	73	13

Anemia	71	13

Seizures, epilepsy or convulsions	60	11

Rapid heart beat or tachycardia	45	8

Gastritis	40	7

Diabetes*	37	7

Emphysema, chronic bronchitis, COPD*	35	6

Ulcer	32	6

Tuberculosis	28	5

Rheumatoid arthritis*	24	4

Peripheral neuropathy	21	4

Heart attack*	19	3

Heart failure	18	3

Stroke*	16	3

HIV*	16	3

Pancreatitis	14	2

Cancer*	13	2

Poor kidney function*	12	2

Cirrhosis	11	2

* Condition included in Katz Comorbidity Index

^1 ^<2% had blood clots in legs or lungs, septic arthritis, endocarditis, atrial fibrillation, cancer (mouth, throat, larynx, esophagus, stomach), peripheral vascular disease*, AIDS*, lupus*, leukemia* or polycythemia vera*, Alzheimer's or other dementia*, lymphoma*, kidney transplant* or polymylagia rheumatica*

Subjects reported a wide range of medical conditions (Table 2). Hepatitis was reported by nearly one third of subjects, the most common single condition. Hypertension was reported by 21% of subjects and about 20% reported asthma. Ten to thirteen percent of subjects reported skin infections, pneumonia, anemia or seizures. Five to ten percent reported tachycardia, gastritis, diabetes, COPD, ulcer, or tuberculosis. Few (2-5%) reported rheumatoid arthritis, neuropathy, heart conditions, stroke, HIV, pancreatitis, cancer, poor kidney function or cirrhosis, and remaining conditions were even more rare.

### Effect of Chronic Disease on Addiction Treatment Utilization

In multivariate regression analyses (Table 3), no significant effect was detected for the main independent variable of chronic disease status on the odds of attending addiction treatment (adjusted odds ratio [AOR] 0.88 for lower severity vs. no chronic disease, 95% confidence interval (CI): 0.60, 1.28; AOR 1.29 for higher severity vs. no chronic disease, 95% CI: 0.89, 1.88). Similarly, no significant effect of chronic disease status was detected on number of outpatient days or residential nights.

**Table 3:** Multivariate Regression of Chronic Disease on Addiction Treatment Utilization (N = 498)

	Model 1	Model 2	Model 3
	Any Addiction Treatment	Outpatient Days	Residential Nights
**Independent Variable**	**AOR (95% CI)**	**IRR (95% CI)**	**IRR (95% CI)**

Chronic disease (Katz)			
None	Referent	Referent	Referent
One, of lower severity only	0.88 (0.60, 1.28)	0.86 (0.41, 1.81)	0.68 (0.31, 1.49)
One of higher severity or multiple conditions	1.29 (0.89, 1.88)	1.14 (0.55, 2.38)	0.63 (0.23, 1.69)

Age	0.99 (0.98, 1.01)	1.00 (0.97, 1.04)	0.99 (0.96, 1.03)

Female	1.23 (0.89, 1.70)	1.74 (0.95, 3.20)	0.68 (0.31, 1.48)

Race			
Black	0.57 (0.38, 0.85)**	0.47 (0.21, 1.05)	0.22 (0.08, 0.64)**
Hispanic	0.77 (0.49, 1.20)	1.34 (0.62, 2.89)	0.55 (0.22, 1.36)
Other	0.61 (0.32, 1.15)	0.75 (0.22, 2.57)	0.77 (0.27, 2.24)
White	Referent	Referent	Referent

Substance dependence			
Alcohol dependent & recent heavy use	0.80 (0.52, 1.24)	0.78 (0.33, 1.83)	1.13 (0.50, 2.57)
Drug dependent & recent use	0.86 (0.61, 1.22)	0.87 (0.39, 1.95)	0.48 (0.21, 1.11)
Both	Referent	Referent	Referent

Recruitment site			
Detoxification program	0.82 (0.57, 1.17)	0.37 (0.19, 0.73)**	1.11 (0.48, 2.57)
Other	Referent	Referent	Referent

Criminal justice involvement (past 3 mo)	0.98 (0.73, 1.31)	1.26 (0.70, 2.27)	1.20 (0.66, 2.18)

Homeless (past 3 mo)	1.07 (0.80, 1.43)	0.95 (0.53, 1.72)	1.06 (0.58, 1.95)

Unemployed (past 3 mo)	1.07 (0.79, 1.45)	1.60 (0.79, 3.24)	0.85 (0.47, 1.54)

Health insurance	2.04 (1.24, 3.36)**	3.06 (0.88, 10.68)	2.54 (0.96, 6.71)

Current smoker	0.89 (0.61, 1.30)	0.79 (0.34, 1.81)	1.48 (0.53, 4.11)

Anxiety (Beck)			
Low	Referent	Referent	Referent
Moderate	1.45 (0.99, 2.14)	1.71 (0.80, 3.66)	2.15 (0.97, 4.75)
Severe	1.77 (1.23, 2.53)**	2.12 (1.02, 4.41)*	2.11 (0.94, 4.75)

Model 1 used logistic regression; Models 2 and 3 used Poisson regression.

*p < .05 **p < .01; Wald chi-square tests, df = 1

For the primary outcome of any addiction treatment utilization (Model 1), being black was a negative factor, and having health insurance and severe anxiety were positively associated with any addiction treatment. Being female and having severe anxiety were positively associated with number of outpatient days, and recruitment from a detoxification facility was a negative factor (Model 2). Being black was a negative factor for residential nights (Model 3).

Similar results were found for the secondary independent variables of asthma and PCS, with neither significantly associated with any of the addiction treatment measures. Fair or poor health status was negatively associated with any addiction treatment and with outpatient addiction treatment, but not with residential treatment. Table 4 summarizes the results for each of the secondary independent variables (from separate regression models) on each of the outcome variables.

**Table 4:** Summary of Other Secondary Multivariate Regression Models, with alternate key independent variables (N = 498)

	Model 1	Model 2	Model 3
	Any Addiction Treatment	Outpatient Days	Residential Nights
**Key Independent Variable**	**AOR (95% CI)**	**IRR (95% CI)**	**IRR (95% CI)**

Asthma	1.05 (0.74, 1.49)	1.03 (0.52, 2.03)	1.03 (0.50, 2.12)

Self-reported health status			
Excellent/Very Good/Good	Referent	Referent	Referent
Fair/Poor	0.67 (0.48, 0.94)*	0.40 (0.20, 0.80)**	0.61 (0.31, 1.21)

Physical Component Score (per 1 point increase)	1.01 (0.99, 1.03)	1.00 (0.97, 1.04)	1.01 (0.97, 1.04)

Model 1 used logistic regression; Models 2 and 3 used Poisson regression. Models duplicate those in Table 3 (controlling for age, gender, race, substance dependence, recruitment from detox, criminal justice involvement, homeless, unemployed, health insurance, smoker, anxiety), solely replacing the chronic disease variable with the secondary variables above.

*p < .05 **p < .01; Wald chi-square tests, df = 1

## Discussion

Despite the potential for interference or facilitation of chronic medical disease on utilization of addiction care, no statistically significant effect was found for chronic disease status on addiction treatment utilization in the prior 3 months, in this cohort of alcohol and drug dependent persons, controlling for sociodemographics, type of substance dependence, recruitment site, current smoking, and severity of recent anxiety. Nor was addiction treatment associated with asthma or physical function and quality of life. These findings held for any addiction treatment and for quantity of treatment services received. Only perceived health status, which is a subjective measure, was associated with less addiction treatment utilization. It is encouraging to note that chronic disease itself may not be a barrier to addiction treatment.

On the other hand, it also appears that chronic medical disease did not increase addiction treatment participation in this cohort. People with chronic disease are often connected to the medical treatment system, but the acute focus of episodic care may not extend to providing assistance or motivation for these patients to access addiction treatment. This may be a missed opportunity for medical providers to encourage their patients to seek addiction treatment given that the self-reported perception of health status was frequently only fair or poor. The lack of a significant positive association between chronic disease and addiction treatment utilization suggests that facilitated access to addiction care is not routinely occurring. Such "reachable moments" [41] for the individual with both chronic disease and addiction are perhaps most likely when formalized linkages between primary care and addiction treatment exist or if the primary care doctor is willing to address addiction with the patient [18,42]. One way to increase these reachable moments is to encourage screening, brief intervention, and referral to treatment approaches in the primary care setting. Opportunities for referral to addiction treatment should increase as the number of medical visits increases. An integrated, longitudinal approach to managing both chronic medical disease and substance dependence is likely to be most beneficial [13,43,44].

It is worthwhile to note that no chronic diseases were reported by two thirds of this sample of substance dependent individuals, somewhat better than an estimate of 53% with no chronic conditions in a comparable population 10 years earlier [22]. The lower estimate here may be due to recruitment beyond detoxification settings; subjects recruited from the community may be less medically ill than those in detoxification facilities. Most of the chronic diseases noted here had prevalence within the range of national and state estimates [45,46]. The asthma prevalence here appears slightly higher than the state average, but this likely reflects the higher rates often found in inner city and African-American populations [47] and the high prevalence of current smoking in this sample. The below average Physical Comorbidity Score in this population is comparable to similar populations 10-15 years earlier [22,48].

Although we did not detect an association between chronic medical variables and treatment utilization, several other variables were significantly related to treatment utilization, including several sociodemographic variables. Health insurance was positively associated with any addiction treatment utilization, demonstrating an expected increase in access to treatment, but did not reach significance for the count variables. Recruitment from detoxification was negatively associated with outpatient days, highlighting the severity of addiction treatment needs for this group, and the expected lack of treatment preceding detoxification entry. Psychiatric comorbidity (e.g., anxiety) was positively associated with addiction treatment utilization reflecting other findings in the literature [49], although this past-week measure was collected for most individuals soon after detoxification, so may reflect short-term anxiety symptoms rather than a chronic disorder.

The lack of findings concerning our primary hypothesis may simply be the reflection of a complex relationship between chronic disease and addiction, with many competing forces. Several opposing effects from chronic disease may have occurred, which could collectively show little or no effect on addiction treatment utilization. First, individuals with chronic disease may be functionally less able to access and participate in addiction treatment, reducing the likelihood of treatment utilization. If physical function were an important barrier, we should have seen some effect from the PCS, which did not occur, but it might be important to consider specific functional limitations. By considering asthma separately, thus removing the variability across conditions, we might have highlighted the functional effects, but that also did not occur. It is also possible that the severity of addiction, as indicated by most individuals in the sample recruited from detoxification, overshadowed any functional limitations as they sought treatment; a desire to focus on the most pressing problem has been highlighted in some literature [9]. Second, for a variety of reasons, addiction treatment facilities may be less likely to accept individuals with medical conditions, who may require medications or have more acute medical needs. This study could not address that question. Third, chronic disease may interfere with the quality of treatment participation; number of visits could be construed as a proxy for this, but if chronic disease affects self-care management this issue may go beyond what could be considered here. Fourth, 3 months is a relatively short time period, so may have masked any differences that would appear with longer history of treatment utilization. However, the focus here is on relative differences in utilization, so the 3 month period should have been sufficient to compare groups. Fifth, and alternatively, individuals with chronic disease may be more likely to access treatment due to their likely existing linkages with the healthcare system. A study of women with trauma and mental or substance use disorders found that greater disability was associated with more outpatient counseling, but not residential, group counseling or peer treatment [50]. While referral from the healthcare system is not a common pathway to addiction treatment [51] the finding that physicians are more likely to know about substance abuse if the patient has an episodic or chronic medical condition [11] suggests that this pathway may be more likely for these patients.

The characteristics of the study sample may have tempered the potential relationships between chronic disease and addiction treatment utilization. In a relatively young sample such as this, chronic disease is less likely, and related problems are likely to be less severe than in an older population. However, addiction itself may increase such problems even in a young sample [22]. The subjects in this study are largely reflective of individuals in addiction treatment, and these results should apply to similar cohorts. Relatively few individuals in this sample had severe chronic disease or multiple conditions, thus findings may be different in an analysis of only individuals with chronic disease. The implications of recruiting much of the sample during a detoxification stay should be considered, where individuals entering detoxification may be unlikely to have been in addiction treatment during a period of substance use severe enough to warrant detoxification. These findings also represent a sample from a single site, so may be less generalizable than recruitment from multiple sites.

A limitation is that the study may have been inadequately powered to detect differences of the observed magnitude. Power calculations estimated 80% power to detect an odds ratio as small as 2.1 for those with higher severity chronic disease, thus it is likely that the study was not adequately powered to detect the small observed magnitude of association of 1.3 (for higher severity). However, since the observed degree of association may still be clinically meaningful, the possibility that individuals with higher comorbidity are more likely to utilize addiction treatment should be further studied in a larger sample to see if a small but clinically important effect is detectable. But the effect of greater concern, interference with receiving addiction treatment due to a chronic disease, is unlikely based on our findings. Last, the current analysis relied on retrospective data from a sample of individuals, most of whom had recently completed detoxification. A prospective or qualitative study would be better able to track the likely causal issues related to chronic disease and addiction treatment access.

To our knowledge, this is the first analysis of the role of chronic disease in addiction treatment utilization. Current household studies on barriers to addiction treatment, such as SAMHSA's National Survey on Drug Use and Health (NSDUH), do not consider medical comorbidities, so are unable to shed light on these underlying questions. Further research in this area is warranted to better understand these complex issues. The individuals in this study were, by definition, willing to receive health care. Other populations may differ. Also, samples with a large number of individuals with chronic disease would allow a focus on more specific chronic diseases, such as diabetes or heart disease, and would allow a better consideration of other psychiatric comorbidity. In the meantime, we highlight the conclusion from these data that chronic disease is not a significant barrier to addiction treatment utilization. While we found no significant association between chronic medical disease and addiction treatment utilization, these issues are complicated and we suggest prospective and qualitative studies to further explore these complexities.

## Competing interests

The authors declare that they have no competing interests.

## Authors' contributions

All authors contributed to the design, interpretation of results, and the manuscript preparation and have approved the final manuscript. In addition, significant individual contributions are as follows. SR conceived of the study, designed the analytic plan, and led the writing, interpretation of results, and revisions of the manuscript. MJL contributed to the study scope, analytic plan, and interpretation of results. DMC provided statistical expertise, and contributed to the study scope, analytic plan, and interpretation of results. DA conducted the statistical analysis. RS and JS lead the parent study from which the data were obtained, brought expertise regarding the data and the study population, and contributed to the study scope, analytic plan, and interpretation of results.
